# Anterosuperior mediastinal paraganglioma in a 42-Year-old woman: a diagnostic and therapeutic challenge—a case report

**DOI:** 10.1186/s13019-024-03283-9

**Published:** 2025-04-07

**Authors:** Zheng Wang, Wenkang Zong, Shuo Liang, Fang Zhou, Xike Lu, Daqiang Sun

**Affiliations:** 1 Department of Thoracic Surgery, Tianjin Chest Hospital Affiliated to Tianjin University, Tianjin, China; 2 Department of Pathology, TTianjin Chest Hospital Affiliated to Tianjin University, Tianjin, China; 3 Department of Radiology, Tianjin Chest Hospital Affiliated to Tianjin University, Tianjin, China; 4 Department of Thoracic Surgery, Tianjin Chest Hospital, No. 261 Taierzhuang South Road, Jinnan District, Tianjin, 300222 China

**Keywords:** Anterosuperior mediastinal paraganglioma, Surgical management, Diagnostic imaging, Multidisciplinary approach, Case report

## Abstract

**Background:**

Paragangliomas are rare neuroendocrine tumors predominantly located within the adrenal gland. Extra-adrenal paragangliomas, particularly those in the anterosuperior mediastinum, are exceedingly rare and pose significant diagnostic and therapeutic challenges due to their complex anatomical location.

**Case description:**

A 42-year-old woman was found to have an anterosuperior mediastinal mass during a routine health screening. Enhanced chest computed tomography (CT) revealed an ovoid, low-density mass intricately associated with major vascular structures including the superior vena cava, brachiocephalic trunk, left common carotid artery, aortic arch, right anonymous vein, and right subclavian artery. Despite significant intraoperative blood loss of 2000 ml, the mass was successfully excised with meticulous surgical technique and effective hemostasis. Histopathological examination showed a classic Zellballen pattern with chief cells and sustentacular cells embedded in a vascular-rich stroma. Immunohistochemistry confirmed the tumor’s chromaffin nature, with chief cells testing positive for CD56, Synaptophysin, and Chromogranin A, and sustentacular cells positive for S100 protein, consistent with a diagnosis of paraganglioma. The patient’s postoperative recovery was uneventful, and she was discharged one week after surgery.

**Conclusions:**

This case highlights the essential role of comprehensive preoperative imaging and the necessity for interdisciplinary surgical expertise in managing complex mediastinal paragangliomas. Advanced surgical techniques and careful intraoperative management are paramount to achieving successful outcomes. Appropriate imaging modalities and auxiliary laboratory tests are crucial for early detection of recurrences in these rare tumors.

## Highlights

Key findings

What is known and what is new?

What is the implication, and what should change now?

• Report here about key findings of the study.

• Report here about what is known.

• Report here about what does this manuscript adds.

• Report here about implications and actions needed.

## Introduction

Pheochromocytomas (PCC) and paragangliomas (PGL), with a combined estimated incidence of 2–8 cases per million person-years, are neuroendocrine tumours originating from chromaffin cell. According to the WHO definition, PCC refers specifically to tumours arising within the adrenal medulla, whereas PGL refers to extra-adrenal tumours. Approximately 10–18% of these tumours occur in extra-adrenal locations, notably within the abdominal and thoracic regions and less commonly in the posterior mediastinum [1]. PGL in the anterosuperior mediastinum are exceedingly rare, contributing to a small fraction of these cases with scant literature documenting their occurrence [2].

These tumours often manifest with sustained arterial hypertension, headaches, and diaphoresis when functional. Conversely, non-functional mediastinal PGL typically present via mass effects rather than catecholamine secretion, leading to symptoms such as chest pain and respiratory difficulties. The surgical removal of anterosuperior mediastinal PGL poses significant challenges due to their proximity to critical vascular structures and heart, compounded by their rich vascularity and adherence to adjacent tissues. The complexity of the surgical approach is often heightened by the necessity for cardiopulmonary bypass due to substantial intraoperative bleeding risks.

Mediastinal PGL are generally resistant to chemotherapy and radiotherapy, making complete surgical excision the cornerstone of treatment. Emerging evidence supports the utility of preoperative embolization in reducing blood loss during surgery, enhancing the safety of tumour resection. The management of these complex cases underscores the importance of meticulous preoperative planning and robust multidisciplinary collaboration.

This report aims to shed light on a rare instance of a 42-year-old woman diagnosed with an anterosuperior mediastinal PGL, underscoring the diagnostic quandaries and surgical hurdles encountered, thereby enriching the discourse on effective management strategies for this uncommon pathology. We present this article in accordance with the CARE reporting checklist.

## Case presentation

**Fig. 1:**
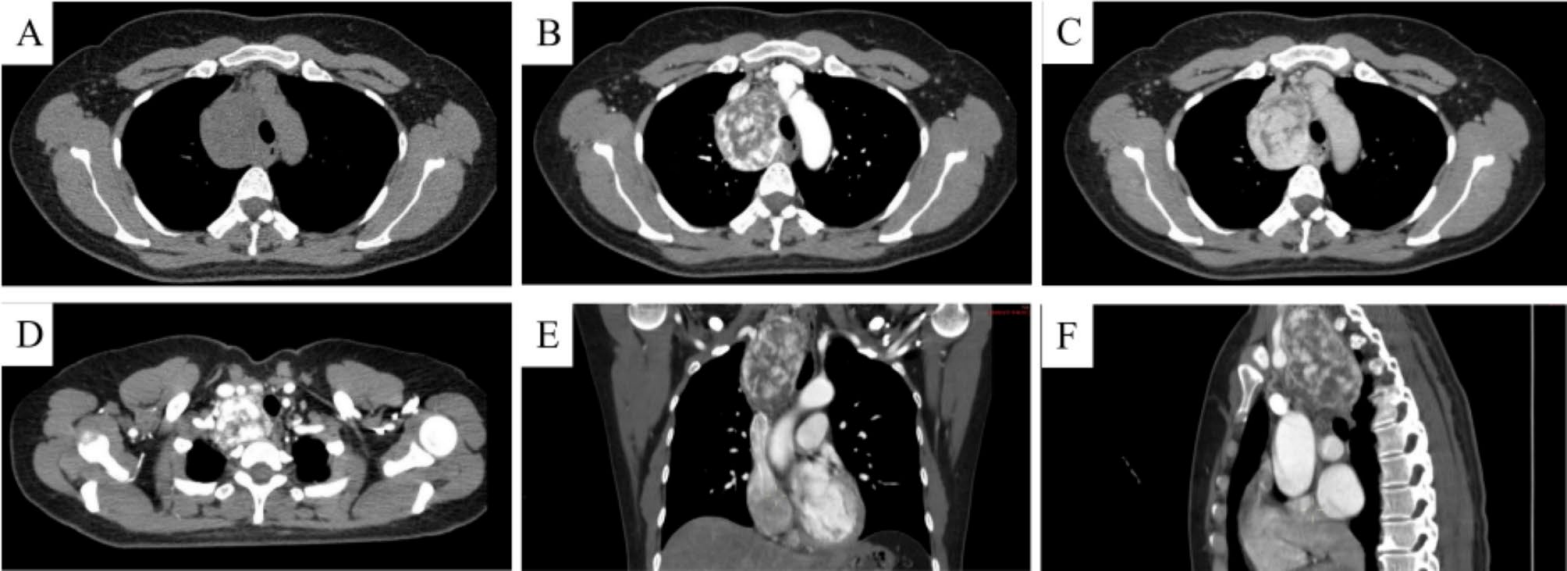
Demonstrates a rounded low-density lesion adjacent to the trachea in the upper mediastinum on the right, measuring approximately 6.5 cm × 4.5 cm, with a CT attenuation of about 34 HU. The trachea is slightly compressed and displaced rightward. The enhancement scan highlights significant enhancement in certain areas, with multiple unenhanced fine linear shadows within. The lesion is intimately associated with the superior vena cava, right brachiocephalic vein, and right subclavian artery. Panel A shows the lesion in non-contrast phase; Panel B during arterial phase with marked enhancement; Panel C illustrates persistent enhancement in the delayed phase; Panels D, E, and F depict the upper boundary of the tumor and reconstructed sagittal and coronal views, respectively

A 42-year-old woman was referred to our centre following the incidental identification of a mediastinal mass during a routine health screening. She reported mild chest discomfort but denied cardinal symptoms, including dyspnoea, cough, or unintentional weight loss. Her medical and family histories were unremarkable. On examination, she appeared well, with stable vital signs and no cardiopulmonary abnormalities. Further imaging with enhanced chest CT revealed an approximately 6.5 cm × 4.5 cm ovoid, heterogeneously enhancing low-density mass located in the anterosuperior mediastinum. This mass exhibited heterogeneous density and was intimately associated with the superior vena cava, brachiocephalic trunk, left common carotid artery, aortic arch, right anonymous vein, and right subclavian artery, showing significant vascular enhancement upon contrast examination. Detailed imaging features are presented in Fig. 1. The preoperative imaging differential diagnosis considered a retrosternal goiter or Castleman’s disease. Laboratory tests indicated a neurone-specific enolase (NSE) level of 58.2 ng/mL, with other parameters within normal limits.

Owing to the tumor’s proximity to major vascular structures, a median sternotomy was performed to provide optimal exposure. The operation required a coordinated effort between cardiothoracic and thoracic surgeons. The cardiac surgeon initiated the dissection using an electrocautery device for precise tissue separation, ensuring minimal thermal damage to surrounding tissues. A suction device was employed simultaneously for blunt dissection, allowing for atraumatic separation of the tumor from adjacent critical structures, including the superior vena cava, brachiocephalic trunk, and right subclavian artery. Branching vessels encountered during this phase were securely ligated using titanium clips to achieve effective haemostasis.

Following the vascular dissection, the thoracic surgeon proceeded with tumor excision. The tumor was found to be highly vascularised and tightly adhered to the surrounding tissues, necessitating a stepwise dissection technique to minimise bleeding. Careful attention was paid to maintaining a clear operative field through continuous suction and intermittent haemostatic measures. Despite the absence of major vascular injury, significant capillary oozing was encountered, resulting in a total blood loss of 2000 mL, which was managed with intraoperative transfusion.

**Fig. 2:**
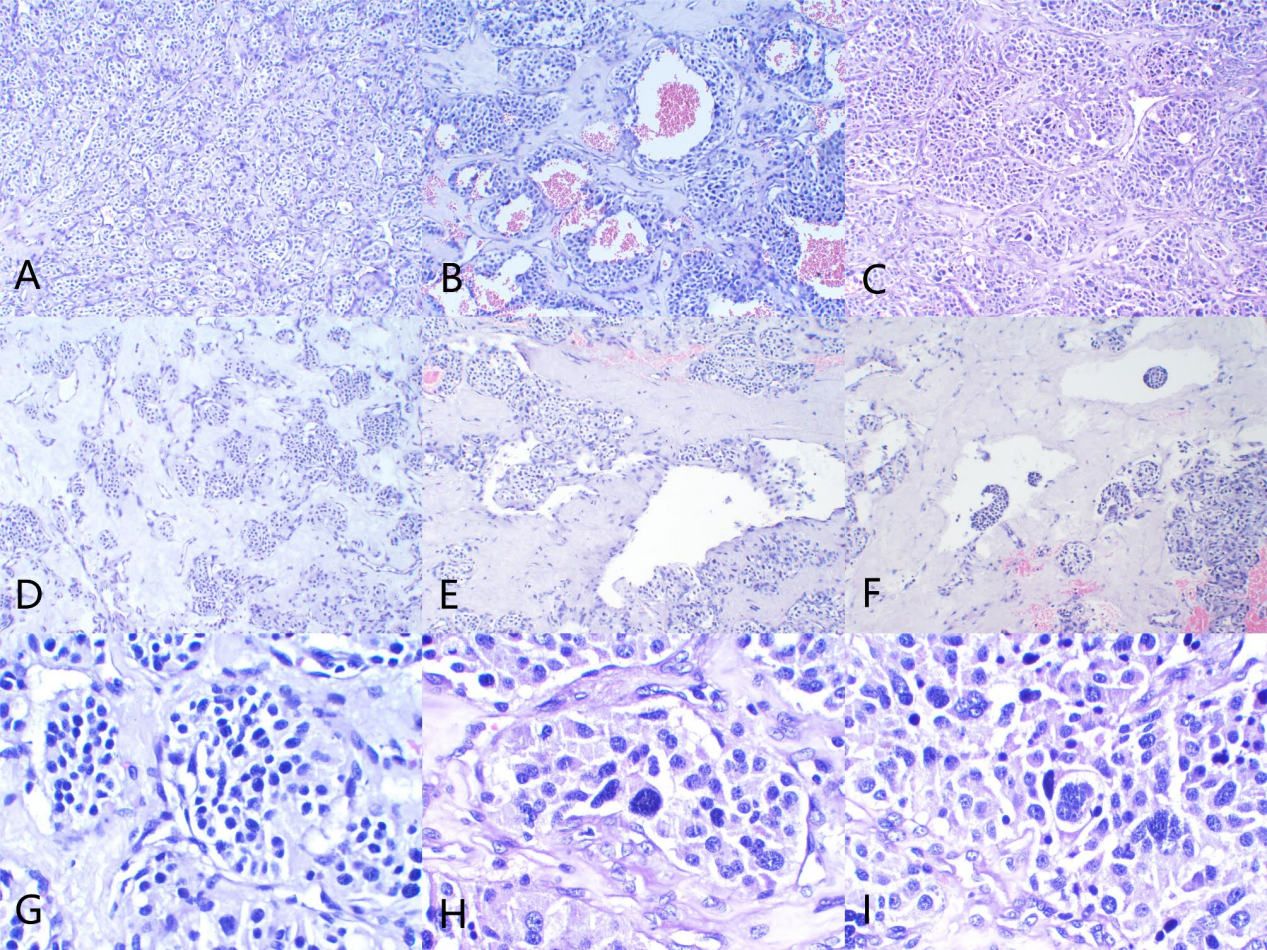
Hematoxylin and eosin (H&E) staining displaying the pathological features of a paraganglioma. Panels A to C show the tumor cells arranged in nests at 100x magnification, with visible hemorrhage and local regions displaying enlarged nucleoli with significant atypia. Panels D to F exhibit hyaline degeneration in the tumor stroma, with tumor invasion into vessel walls and intravascular tumor thrombus at 100x magnification. Panels G to I at 400x magnification reveal nests of cells with notably enlarged nucleoli and atypia, including visible tumor giant cells

Pathological examination of the resected mediastinal mass from the same patient revealed irregular tissue measuring 7 × 5 × 2.5 cm, with a capsule-like surface and a cut surface that was grayish-red and pinkish-gray, solid, and of medium consistency. Under low magnification, the tumor cells were arranged in a nesting pattern (Zellballen), with some areas exhibiting diffuse and trabecular patterns. The stroma was rich in thin-walled blood sinusoids forming a complex vascular network, with some areas showing fibrous connective tissue proliferation and hyalinization. High magnification revealed that the tumor consisted of chief cells and sustentacular cells; chief cells were round or polygonal, occasionally spindle-shaped, with abundant pale eosinophilic cytoplasm and occasional clear cells, and small round to oval nuclei. Sustentacular cells were fewer, smaller, and primarily distributed around the cell nests. Mitotic figures were rare. As shown in Fig. 2, Panels A to C illustrate the tumor cells arranged in nests (Zellballen pattern) at 100x magnification, with visible hemorrhage and regions displaying enlarged nucleoli with significant atypia. Panels D to F demonstrate hyaline degeneration in the tumor stroma, with tumor invasion into vessel walls and intravascular tumor thrombus, also at 100x magnification. Panels G to I, at 400x magnification, reveal nests of cells with notably enlarged nucleoli and atypia, including visible tumor giant cells, providing detailed cellular characteristics.

**Fig. 3:**
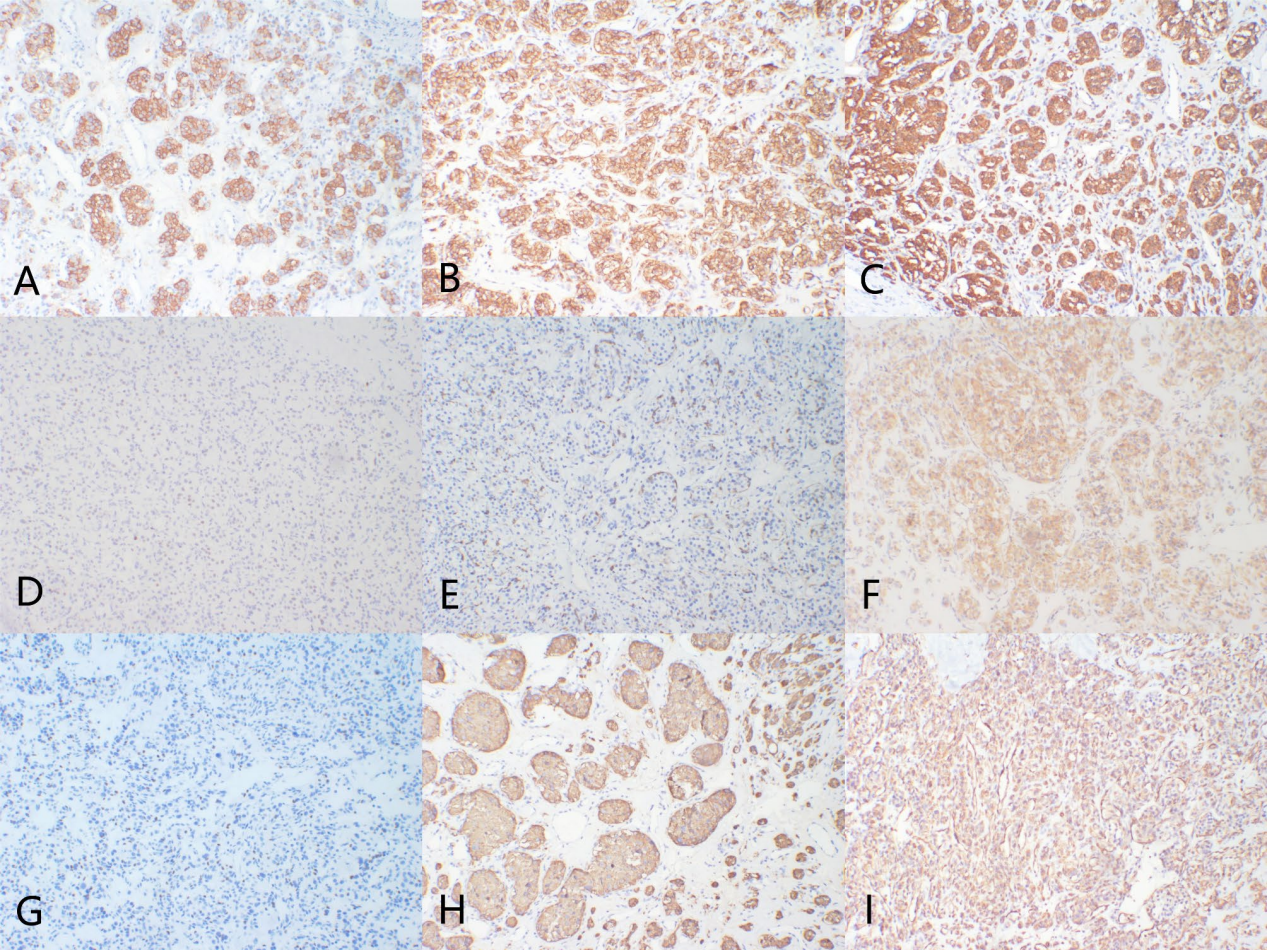
Immunohistochemical staining delineates protein expression within the tumor cells (all at 100x magnification). Panel A demonstrates strong positive expression of CD15; Panel B of CD56; Panel C of Chromogranin A (CgA); Panel D shows scattered positive expression of GATA3; Panel E positive expression of S100 in supporting cells; Panel F strong expression of Succinate Dehydrogenase Complex Subunit B (SDHB); Panel G positive expression of SOX10 in supporting cells; Panel H of Synaptophysin (Syn); and Panel I shows Vimentin positive expression in some cells, indicating its stromal origin

Immunohistochemical staining of the same patient’s tumor showed positivity for CD56, Synaptophysin, Chromogranin A, CD15, and SDHB in the chief cells, with scattered positivity for GATA3 and negativity for cytokeratin; sustentacular cells were positive for S100 and SOX10. These findings led to a final diagnosis of paraganglioma. As shown in Fig. 3, Panel A demonstrates strong positive expression of CD15, indicating the presence of this marker in the tumor cells. Panel B shows positivity for CD56, confirming the neuroendocrine nature of the tumor. Panel C reveals strong expression of Chromogranin A (CgA), a classic marker for neuroendocrine tumors. Panel D displays scattered positive expression of GATA3, while Panel E highlights positive expression of S100 in supporting cells, indicative of the presence of sustentacular cells. Panel F demonstrates strong expression of Succinate Dehydrogenase Complex Subunit B (SDHB). Panel G shows positive expression of SOX10 in supporting cells. Panel H indicates positivity for Synaptophysin (Syn), and Panel I shows Vimentin positive expression in some cells, indicating its stromal origin. The patient recovered well postoperatively and was discharged a week after surgery. At the last follow-up, conducted two weeks postoperatively during an outpatient visit, the patient underwent suture removal. A chest X-ray performed at that time showed changes consistent with the thoracotomy and elevation of the right hemidiaphragm, likely attributable to phrenic nerve injury sustained during surgery. No other complications or signs of recurrence were observed at this follow-up.

All procedures performed in this study were in accordance with the ethical standards of the institutional and/or national research committee(s) and with the Declaration of Helsinki (as revised in 2013). Written informed consent was obtained from the patient for publication of this case report and accompanying images. A copy of the written consent is available for review by the editorial office of this journal.

## Discussion

This case report describes a rare instance of a 42-year-old woman with an anterosuperior mediastinal paraganglioma, a neuroendocrine tumor originating from non-adrenal sympathetic or parasympathetic chromaffin cells. Although most paragangliomas are incidentally diagnosed or identified due to mass-related symptoms, functional mediastinal paragangliomas are exceedingly rare, with an incidence of less than 2%. These tumors typically do not present with the classic symptoms of catecholamine excess, such as hypertension, palpitations, or sweating, making their diagnosis particularly challenging in the absence of overt symptoms.

The Mayo Clinic conducted a retrospective study from 2000 to 2015 on patients undergoing thoracic PGL excision, revealing that 73% of the 22 patients had functional tumors [3]. However, in our case, the patient was asymptomatic, and the tumor was initially mistaken for a retrosternal thyroid goiter or Castleman’s disease due to a lack of specific catecholamine-related testing.

Historically, the first description of paragangliomas dates back to 1866 by Felix Fraenkel. The diverse biological behavior of these tumors may be influenced by their genetic background and anatomical location. According to the latest WHO classification, paragangliomas within the adrenal gland are considered sympathoadrenal paragangliomas, while those occurring outside exhibit a broader origin and phenotype, aiding clinicians in treatment decision-making [4].

In imaging studies, paragangliomas typically appear as highly vascularized masses on CT scans, often located at major vascular bifurcations, and can present with either homogeneous or heterogeneous enhancement [5]. A study involving 51 mediastinal paragangliomas indicated that the majority were located in the middle mediastinum (71%), followed by anterior (including anterosuperior) (18%) and posterior mediastinum (12%). In our case, the initial misdiagnosis was influenced by the tumor’s radiological appearance, which closely mimicked conditions such as retrosternal goiter (due to the tumor’s location reaching above the thoracic inlet adjacent to the thyroid) or Castleman’s disease (due to its location at the right side of the upper mediastinum near lymph nodes).

The definitive diagnosis of paraganglioma relies heavily on pathological examination due to the non-specific nature of imaging characteristics. The histological features of paragangliomas include high vascularity and the presence of acidophilic cell clusters surrounded by numerous capillaries. The positive immunohistochemical staining results are crucial for diagnosis. Histopathological scoring systems like PASS and GAPP provide frameworks for assessing the malignant potential of paragangliomas by evaluating histological features and immunohistochemical markers such as SDHB. Succinate dehydrogenase (SDH) mutations, particularly SDHB mutations, are known to be more common in mediastinal paragangliomas compared to those outside the mediastinum. This is a significant finding that should be considered in the diagnostic evaluation of mediastinal PGLs. In this case, the size of the tumor, its invasive microscopic features, and its relationship to significant anatomical structures indicated a potentially high-risk and complex scenario.

In terms of treatment, although complete surgical excision of paragangliomas presents challenges due to their highly vascular nature, it remains the preferred method due to its potential to significantly improve long-term survival rates. Due to their highly vascularized nature, mediastinal paragangliomas pose a significant risk of hemorrhagic complications during biopsy or needle-based diagnostic procedures. Consequently, surgery is not only the preferred treatment but also the primary means of obtaining a definitive diagnosis through postoperative pathology. The successful treatment of our case relied on accurate preoperative assessment, sophisticated surgical techniques, and the close collaboration between cardiothoracic and thoracic surgeons, highlighting the importance of a multidisciplinary approach in managing such complex clinical cases.

## Conclusions

In summary, while anterosuperior mediastinal paragangliomas are rare and can mimic other tumors such as retrosternal goiter, their challenging surgical excision primarily stems from their high vascularity. Therefore, when encountering a mediastinal mass, inclusion of paraganglioma in the differential diagnosis is imperative.

## Data Availability

No datasets were generated or analysed during the current study.
